# Therapeutic potential of pregnenolone and pregnenolone methyl ether on depressive and CDKL5 deficiency disorders: Focus on microtubule targeting

**DOI:** 10.1111/jne.13033

**Published:** 2021-09-08

**Authors:** Isabella Barbiero, Massimiliano Bianchi, Charlotte Kilstrup‐Nielsen

**Affiliations:** ^1^ Department of Biotechnology and Life Sciences, (DBSV) Centre of NeuroScience University of Insubria Busto Arsizio Italy; ^2^ Ulysses Neuroscience Ltd. Trinity College Dublin Dublin Ireland; ^3^ Institute of Neuroscience Trinity College Dublin Dublin Ireland

**Keywords:** CDKL5 deficiency disorder, major depressive disorders, microtubules, pregnenolone‐methyl‐ether

## Abstract

Pregnenolone methyl‐ether (PME) is a synthetic derivative of the endogenous neuroactive steroid pregnenolone (PREG), which is an important modulator of several brain functions. In addition to being the precursor of steroids, PREG acts directly on various targets including microtubules (MTs), the functioning of which is fundamental for the development and homeostasis of nervous system. The coordination of MT dynamics is supported by a plethora of MT‐associated proteins (MAPs) and by a specific MT code that is defined by the post‐translational modifications of tubulin. Defects associated with MAPs or tubulin post‐translational modifications are linked to different neurological pathologies including mood and neurodevelopmental disorders. In this review, we describe the beneficial effect of PME in major depressive disorders (MDDs) and in CDKL5 deficiency disorder (CDD), two pathologies that are joint by defective MT dynamics. Growing evidence indeed suggests that PME, as well as PREG, is able to positively affect the MT‐binding of MAP2 and the plus‐end tracking protein CLIP170 that are both found to be deregulated in the above mentioned pathologies. Furthermore, PME influences the state of MT acetylation, the deregulation of which is often associated with neurological abnormalities including MDDs. By contrast to PREG, PME is not metabolised into other downstream molecules with specific biological properties, an aspect that makes this compound more suitable for therapeutic strategies. Thus, through the analysis of MDDs and CDD, this work focuses attention on the possible use of PME for neuronal pathologies associated with MT defects.

## Graphical abstract

Pregnenolone methyl‐ether (PME) is a synthetic derivative of the endogenous neuroactive steroid pregnenolone (PREG). PREG and PME promote microtubule dynamics by possibly affecting the microtubule‐binding of MAP2 and the plus‐end tracking protein CLIP170 with possible therapeutic potential for major depressive disorders and CDKL5 deficiency disorder, both characterised by altered microtubule dynamics.

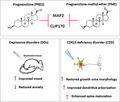

## 1 INTRODUCTION: PREGNENOLONE AND PREGNENOLONE‐METHYL‐ETHER

**FIGURE 1:**
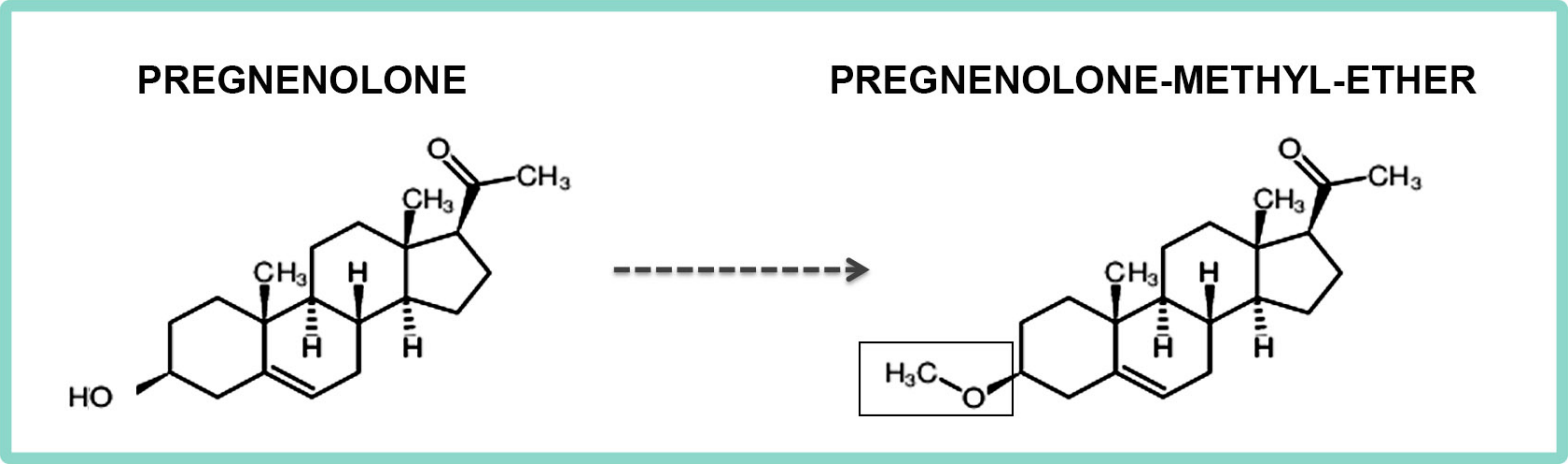
Chemical structure of pregnenolone and pregnenolone‐methyl ether

Pregnenolone (PREG) is an endogenous steroid resulting from the conversion of cholesterol by the mitochondrial enzyme CYP11A1, a process that is conserved in animals from amphibians to mammalians.^1^ PREG is the precursor of most steroid hormones, including the progestogens, androgens, oestrogens, glucocorticoids and mineralocorticoids.^2^ In humans, PREG is converted into its sulphated derivative (PREG‐S), progesterone and 17‐hydroxy PREG; the latter is not present in rodents.^3^ PREG‐S is a neuroactive steroid binding to both GABA_A_ and NMDA type of glutamate receptors.^4^ PREG itself lacks any GABA_A_ and NMDA receptor modulatory activity, although it is a neuroactive steroid and its biological targets have only recently been unravelled.^5^ Thus, identified targets for PREG include Sigma1 receptors,^6^ pregnane X receptor,^7^ type‐1 cannabinoid receptor and microtubule‐associated proteins (MAPs) such as MAP2^8^ and CLIP170.^9^ Importantly, diminished brain levels of PREG have been found in different brain disorders associated with synaptic pathology, such as psychiatric and neurodegenerative disorders.^10^, ^11^, ^12^ The use of PREG as a therapeutic agent has been explored from the 1940s onward^13^ and, currently, both preclinical and clinical studies suggest neuroprotective effects, which might be beneficial for the treatment of chronic pain, neuropsychiatric disease, drug addiction, neurodegeneration and neurodevelopmental disorders. However, its poor bioavailability, short biological half‐life, and rapid *in vivo* metabolism with conversion into active or inactive steroids and metabolites represent a limitation for using PREG in the clinic. For example, oral administration of PREG in humans results in the production of multiple metabolites, including allopregnanolone and 5β metabolites, which can be detected in biological fluids.^14^, ^15^ Some synthetic PREG analogues have improved bioavailability and efficacy and a safer profile compared to their parent molecule PREG.^5^ Their use, as an alternative to PREG, for the treatment of the diseases indicated above is an active field of research in neuroscience drug discovery. Pregnenolone‐methyl‐ether (PME, 3β‐methoxy‐pregenolone) is a C3 analogue of PREG, which is commercially available and was first synthesised in the 1950s by the American chemist Max Neil Huffman.^16^ PME is methylated in position 3 (Figure 1) and therefore cannot be converted back to PREG or into other steroids and metabolites either peripherally or centrally.^17^ PME rapidly crosses the blood‐brain barrier as shown by a pharmacokinetics study that detected PME in the rat brain following a single injection at 12 mg kg^‐1^, s.c.^17^ PME was screened for *in vitro* binding to 80 different neurotransmitter receptors (including NMDA and GABA_A_) or transporters and no significant affinity was found for any of them.^17^ However, PME keeps the PREG biological activity on MAP2^18^ and CLIP170^19^ and therefore on the modulation of microtubule (MT) dynamics and neuronal plasticity. The current review focuses on the therapeutic potential of PME and its biological activity on the cytoskeletal MAPs. Particular emphasis is given to current state of research for using PME as a treatment for depression and CDKL5 deficiency disorder (CDD).

## 2 MOLECULAR TARGETS OF PME: FOCUS ON THE MT SYSTEM

The MT system is a target of PME that modulates MT dynamics by influencing (i) the α‐tubulin post‐translational modifications (PTMs) and (ii) the activity of specific MAPs. In particular, PME has been shown to target MAP2, which together with Tau and MAP‐4, belongs to the MAP2/Tau family,^20^ and CLIP170, belonging to the family of plus‐end tracking proteins (+TIPs).^21^ The regulation of α‐tubulin PTMs and MAPs is fundamental for the maintenance of neuronal homeostasis and the perturbation of this system can lead to a range of dysfunctions that are linked to human diseases, such as neuropsychiatric and neurodevelopmental disorders.^22^, ^23^

### 2.1 The MT system

MTs are one of the major cytoskeletal components present in all eukaryotic cell types. They are composed of repeated dimers of α‐ and β‐tubulin, forming typically 13 linear protofilaments that associate laterally to create the polarised MT with so‐called plus‐ and minus‐ends. MTs actively modify their structure through dynamic cycles of polymerisation (growth) and depolymerisation (shrinkage). The MT plus‐end can rapidly switch between these two phases, going from growth to shrinkage (catastrophe), or from shrinkage to growth (rescue); this process is called “dynamic instability” or “microtubule dynamics”.^24^

**FIGURE 2:**
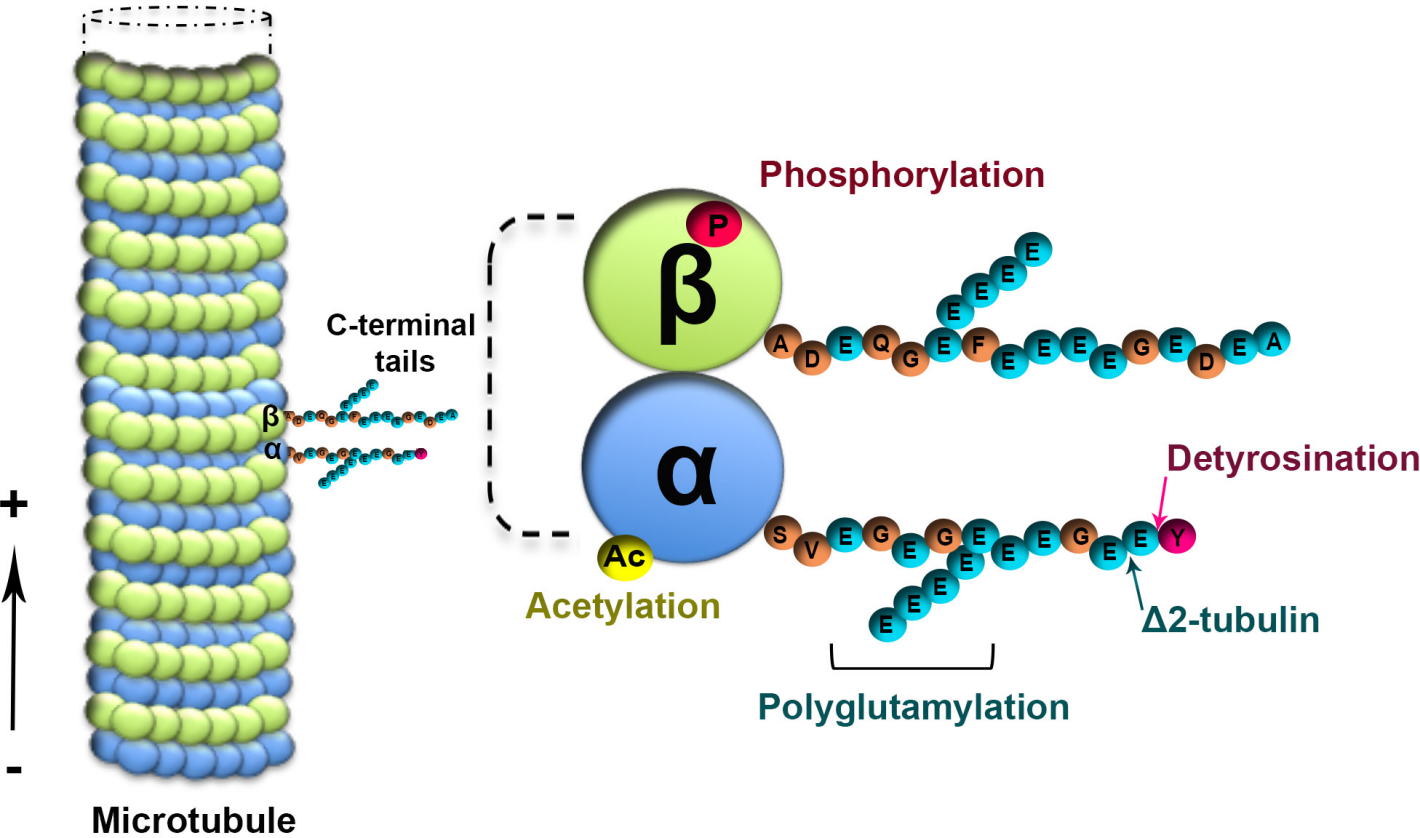
Schematic representation of some different tubulin post‐translational modifications. Microtubules (MT) are dynamic structures that assemble from heterodimers of α‐ and β‐tubulin in a polarised fashion (from the minus‐end towards the plus‐end). Acetylation (Ac) and phosphorylation (P) occur in the inner side of the MT lattice, whereas poly‐glutamylation, tyrosination/detyrosination and C‐terminal deglutamylation (which generates Δ2‐Tub) take place within the C‐terminal tubulin tails that project away from the polarised MT surface

A correct organisation and remodelling of the MT network is essential for neurones to develop axons and dendrites and to form synapses. Moreover, in mature neurones, MTs continue to maintain the cellular architecture and serve as tracks for intracellular trafficking, allowing motor proteins to deliver specific cargoes within the cell.^25^ The neuronal MT network is highly heterogeneous and finely regulated by the presence of multiple tubulin isotypes, which are subjected to PTMs that altogether constitute the so called “tubulin code”. PTMs affect the C‐termini of both α‐ and β‐tubulin subunits. One of these modifications is the tyrosination cycle that involves the removal and the re‐addition of the C‐terminal tyrosine residue of α‐tubulin (Figure 2). This alternation generates tyrosinated α‐tubulin (Tyr‐Tub) and detyrosinated α‐tubulin (Glu‐Tub), which are, respectively, a hallmark of instable and stable MTs. A fine regulation of the Tyr/Glu‐Tub ratio is important during axonal development and transport.^26^ The removal of the last C‐terminal Glu residue generates Glu‐Tub the functional role of which remains poorly understood. α‐tubulin is also acetylated *(*Acet‐Tub), which is a characteristic feature of stable (i.e., less dynamic) MTs. Decreased Acet‐Tub levels have been found in the hippocampus of socially isolated rats^27^ and in *ULK4*‐knockdown cells, a gene associated with schizophrenia.^28^ By contrast, increased Acet‐Tub levels have been observed in rats exposed to acute subchronic and mild chronic stress.^29^, ^30^

Thus, tubulin PTMs generate specific MT subnetworks that are crucial to define neuronal features depending on the stage of neuronal development and the subcellular compartment. For example, the growth cone of immature neurones is mainly composed of dynamic MTs, whereas MTs are extremely stable in axons and dendrites.^31^ More mature neurones, instead, are characterised by dendritic spines, the functional maturation of which is associated with the invasion of dynamic MTs.^32^, ^33^, ^34^, ^35^

### 2.2 MAP2

**FIGURE 3:**
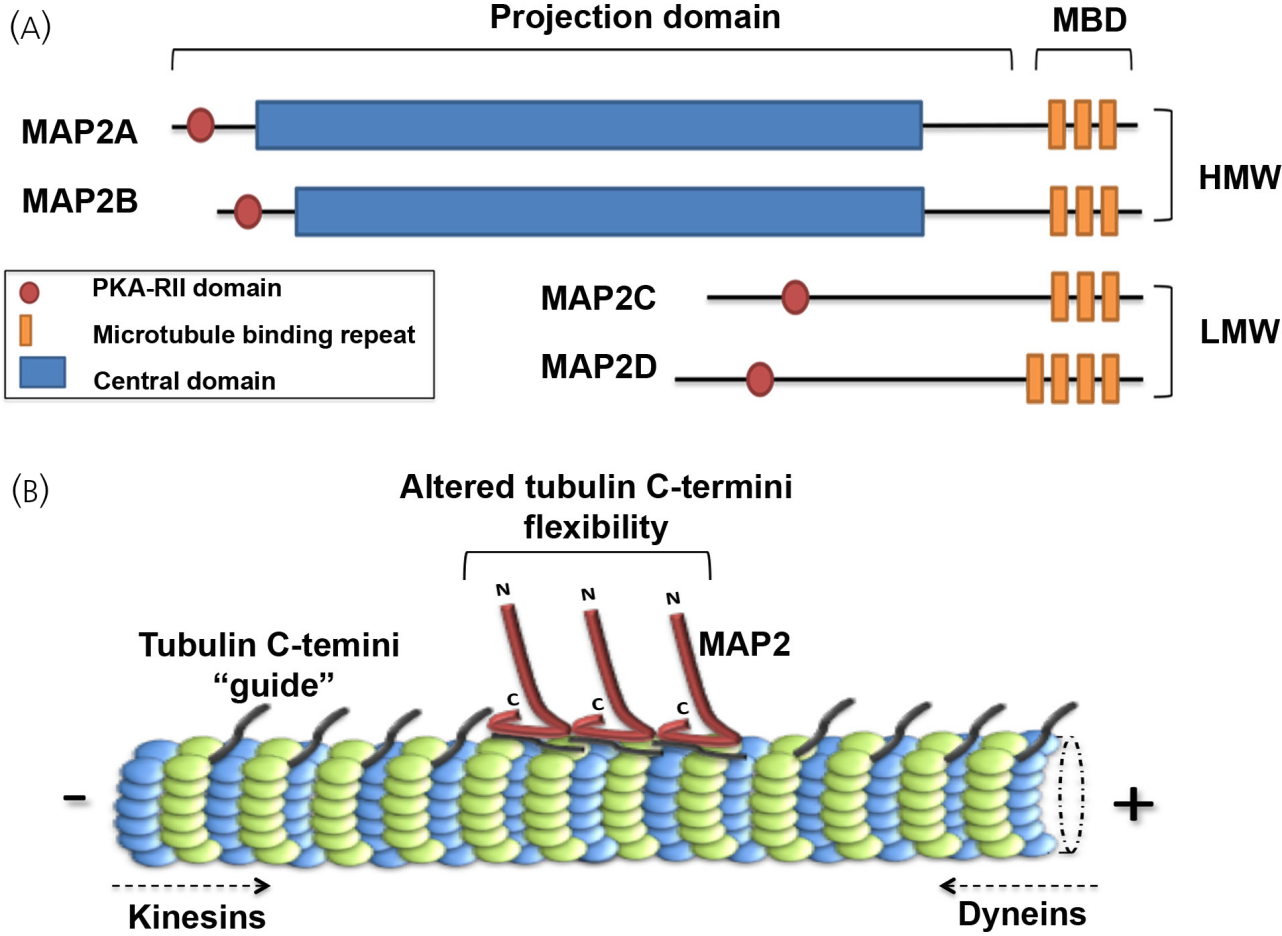
Schematic representation of MAP2 isoforms and their microtubule (MT)‐binding. A, The *MAP2* gene encodes four major transcripts producing four proteins: MAP2A and MAP2B, which belong to the high molecular weight subfamily (HMW), and MAP2C and MAP2D, which represent the lower molecular weight forms (LMW). All forms of MAP2 share a common core sequence which includes the MT‐binding domains (MBD), composed of three or four MT‐binding repeats (18 amino acids long) and a projection domain of various length that contains the interaction domain for the RII subunit of protein kinase A (PKA). Finally, MAP2‐HMW isoforms present a central domain that is missing in the LMW isoforms. B, MAP2 binds the C‐termini of tubulin probably altering its flexibility; this conformational change may therefore modify the processivity of the motors proteins kinesins and dyneins, which govern anterograde (toward MT‐plus end) and retrograde (toward MT‐minus end) transport, respectively

The *MAP2* gene encodes for four isoforms that can be divided according to their molecular size into two groups: high molecular weight MAP2 (HMW‐MAP2, approximately 280 KDa), which includes MAP2A and MAP2B, and the lower molecular weight isoforms (LMW‐MAP2, approximately 70 KDa), including MAP2C and MAP2D. Each protein contains a MT‐binding domain (MBD), and an N‐terminal domain of varying length (projection domain) that includes the regulatory binding region of protein kinase A (PKA‐RII). Additionally, HMW‐MAP2 is characterised by a central domain (CD) that is absent in the LMW‐MAP2 isoforms^20^ (Figure 3).

HMW‐MAP2 is exclusively expressed in neurones where it localises in dendrites and in the soma, whereas it is almost absent in axons.^36^ LMW‐MAP2 is present in every neuronal compartment and, in contrast to HMW‐MAP2, can also be detected in some non‐neuronal cells such as oligodendrocytes.^36^

MAP2 binds MTs longitudinally stabilising tubulin‐tubulin interactions along the protofilaments, which promotes the stabilisation of MTs^37^, ^38^, ^39^ (Figure 3B). The activity of MAP2 is finely regulated by events of phosphorylation and dephosphorylation that are responsible of its association with MTs and/or other MAPs^40^; in the brain, this cycle is often a consequence of activity‐dependent activation of different signalling pathways. Finally, MAP2 has been demonstrated to play a role in neuronal transport by controlling the MT‐association of the kinesin and dynein motor proteins.

The binding of MAP2 to MTs is fundamental for neuronal development and activity. The overall ablation of MAP2 expression or the deletion of the MAP2 MT‐binding domain results in decreased MT density and impaired dendrite elongation, whereas its overexpression leads to increased dendrite number and length.^41^, ^42^, ^43^ Loss of MAP2 has been associated with schizophrenia,^44^ major depressive disorder (MDD)^45^, ^46^ and social isolation.^47^

MT‐binding experiments have demonstrated that the neurosteroid PREG binds MAP2 with high affinity.^18^ Even though the precise binding site of PREG on MAP2 has never been described so far, different studies indicate that PREG is able to bind both HMW and LMW MAP2 isoforms.^8^, ^9^ By contrast, even if PME has been found to promote MT polymerisation like PREG, there is no evidence showing its direct binding to MAP2.^18^ Furthermore, PREG and PME promote the neurite localisation of MAP2 in concomitance with the increase of neurite extension in NGF‐treated PC12 cells. Interestingly, this stimulatory effect is suppressed upon MAP2 silencing, suggesting that MAP2 could indeed be considered an endogenous target for both the compounds.^18^

### 2.3 CLIP170

**FIGURE 4:**
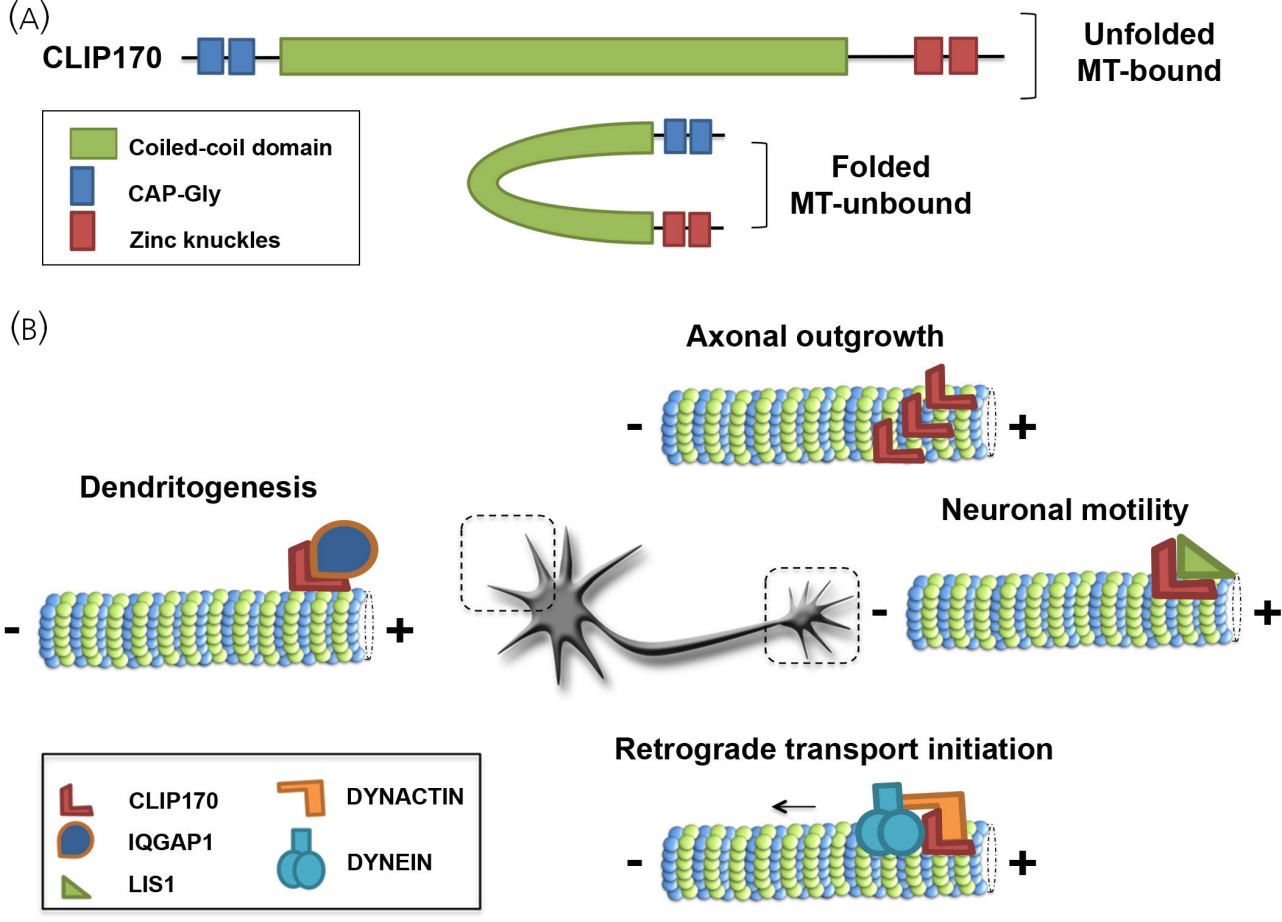
Schematic representation of CLIP170 and its neuronal functions. A, CLIP170 contains two CAP‐Gly motifs at its N‐terminal followed by a long coiled‐coil structure and two putative metal‐binding domains (or zinc knuckles) at the C‐terminal region. The intramolecular interaction of its terminal domains produces the folded microtubule (MT)‐unbound conformation of the protein. B, The open MT‐bound conformation of CLIP170 allows the protein to interact with its binding partners and coordinate some neuronal functions such as axonal outgrowth, neuronal motility, retrograde transport initiation and dendritogensis

Cytoplasmic linker proteins (CLIPs) belong to the family of +TIPs that participate in several neural developmental processes contributing to neuronal morphogenesis^48^ and mediating neuronal motility.^49^ In particular, CLIP170 is composed of two functional domains at the N‐ and C‐termini, separated by a long coiled‐coil region. The N‐terminal region cytoskeleton‐associated protein glycine‐rich (CAP‐Gly) motifs mediate the association of the protein to MTs. The C‐terminal metal‐binding motifs, the zinc knuckles, are able to interact with the N‐terminal region through an intramolecular interaction that generates a closed auto‐inhibited conformation interfering with the MT‐binding of CLIP170^50^ (Figure 4). In the extended conformation, CLIP170 operates as a rescue factor that converts shrinking MTs to growing ones. Importantly, the switch between its folded and unfolded conformation is mediated by phosphorylation events that occur on multiple residues and, according to the specific phosphorylation site, can either enhance or reduce the affinity of CLIP170 for MTs.^50^, ^51^, ^52^ Among the CLIP family members, CLIP115 that lacks the C‐terminal zinc knuckles also exists, which precludes intramolecular folding.^53^

As mentioned, CLIP170 is expressed in the developing nervous system^33^; it forms comet‐like structures in the cell body and in the neurites of expressing neurones.^54^ Furthermore, it is enriched in the axonal growth cone where it positively regulates MT dynamics, thereby promoting axon formation. Indeed, the expression of a dominant negative mutant of CLIP170 prevents the MT‐binding of the endogenous protein causing a destabilisation of the MT network in the growth cone and thus interfering with axonal outgrowth.^54^ CLIP170 is also implicated in the regulation of neuronal motility through the interaction with LIS1, which is associated with lissencephaly 1.^49^ Additionally, CLIP170 cooperates with the scaffolding protein IQGAP1 for the proper establishment of dendritic arborisation.^55^ Finally, the activity of CLIP170 is required for the initiation of retrograde dynein‐mediated transport from the distal axon toward the soma through the interaction with the p150^Glued^ subunit of dynactin.^50^, ^56^ The initiation zone of retrograde transport in the distal axon coincides with a region enriched in Tyr‐Tub,^56^, ^57^ which promotes the MT‐binding of CLIP170.

Importantly, a next generation sequencing study of consanguineous Iranian families affected by intellectual disability (ID), led to the identification of a novel loss of function mutation in *CLIP1* (encoding CLIP170 in humans)^58^ suggesting that loss of CLIP1 function can lead to cognitive impairments.

Through the use of PREG‐photoaffinity probes, it was elegantly demonstrated that PREG directly binds CLIP170 inducing its open active conformation.^9^ PREG‐binding would reduce the curvature of the coiled‐coil motif of CLIP170, thus preventing the association between the two extremes of the protein. The cooperation between PREG and CLIP170 promotes cellular migration and increases MT polymerisation. Furthermore, PREG increases the affinity of CLIP170 for its partners p150^Glued^ and LIS1.^9^

Regarding PME, no experiments have yet confirmed its direct interaction with CLIP170. However, recent studies performed in our laboratory demonstrated that PME, similar to PREG, restores the activity of hypo‐functional CLIP170 in *CDKL5*‐silenced cells.^19^ *CDKL5* (cyclin‐dependent kinase‐like 5) encodes for a serine‐threonine kinase, the mutations of which are linked to CDD, a severe neurodevelopmental pathology.^59^ We found that the absence of CDKL5 impacts the binding of CLIP170 to MTs; the treatment of CDKL5 deficient cells with PME and PREG ameliorates such a defect, suggesting an analogous mechanism of action of the two molecules.^19^

## 3 THERAPEUTIC POTENTIAL OF PME IN MDDS

MDDs are defined as a persistent low mood, anhedonia and an abnormal inhibition of functions, such as sleep and concentration, for a period of at least 2 weeks.^60^ The World Health Organisation estimated that, in 2015, approximately 322 million people suffered from depression worldwide,^61^ meaning that more efficacious and safer treatments are urgently needed. This is now even more relevant as a result of the increased rate of MDDs caused by the COVID‐19 pandemic. Thus, a recent study showed a current prevalence of depression of 25%, which is seven times higher compared to a global estimated prevalence of depression of 3.44% in 2017.^62^ Preclinical evidence in rodents suggests that PREG administration has antidepressant‐like^63^ and anxiolytic‐like effects.^64^ Clinical data are so far conflicting. A first clinical trial investigating therapeutic effects of 4 weeks administration of PREG in healthy‐volunteers revealed no improvement in mood.^65^ However, a more recent clinical study suggests that PREG therapy for 12 weeks may improve depressive symptoms in patients with bipolar disorder.^66^ Furthermore, decreased levels of PREG have been found in the cerebrospinal fluid of patients affected by anxiety and depressive disorder, suggesting a defective neurosteroidogenesis in the etiophatogenesis of such disorders.^67^

PME was first employed as a treatment for rheumatoid arthritis; patients had no improvement of the condition but reported a feeling of “wellbeing” associated with an apparent reduction in anxiety, as described by Sleeper.^68^ Therefore, the effects of PME were investigated in psychiatric conditions and improved mood, reduced anxiety and a restored sleep cycle were observed in depressed patients with no major side effects associated to the treatment.^68^ However, research in PME for the treatment of depression did not advance in those years. The reasons for this are unclear, although it should be noted that the use of tricyclic antidepressants as efficacious pharmacological treatments for MDD incresaed in the same period. Thus it might have been difficult to secure funding to support the advancement of steroid‐derivative in the clinic for the treatment of MDD. Later, it was shown that PME exerts its biological activity by interacting with MAP2 to promote MT dynamics and therefore neuronal plasticity^18^ (see also above). MAP2 has been reported decreased in the hippocampus in models of depression in rodents^30^, ^47^ and in post‐mortem samples from depressed patients.^46^ Consistently with these changes in MAP2, MT dynamics was also found affected in animal models of depression as reflected by altered expression of α‐tubulin PTMs such as the Tyr/Glu‐Tub ratio and Acet‐Tub in the hippocampus.^27^, ^29^, ^47^, ^69^, ^70^ Importantly, chronic treatment with antidepressant drugs such as selective serotonin reuptake inhibitors^47^, ^71^ and agomelatine^72^ changes hippocampal expression and the Tyr/Glu‐Tub ratio and Acet‐Tub, indicating a link between MT dynamics and the treatment of depression. Bianchi et al^73^ proposed pregnenolone‐derivatives as potential antidepressant drugs with a novel mechanism of action based on the modulation of MT dynamics. Accordingly, preclinical studies have shown antidepressant‐like efficacy of PME administration in animal models of depression, such as social isolation in rats^74^ and psychosocial stress in tree shrews.^69^ Specifically, PME intraperitoneal treatment (10 mg kg^‐1^, once a day for 7 days) recovered the decrease in the Tyr/Glu‐Tub ratio and increased Acet‐Tub expression induced by social isolation in rat hippocampus together with rapid antidepressant‐like, anxiolytic and pro‐cognitive efficacy.^74^ PME oral administration (50 mg kg^‐1^, once a day for 4 weeks) to tree shrews recovered the stress‐induced decrease in the Tyr/Glu‐Tub ratio and Acet‐Tub accompanied by the rescue of avoidance behaviour, hormone hypersecretion, hypothermia and sleep disturbances.^69^ These results further strengthen the initial observations made in the 1950s regarding a potential anti‐depressant efficacy of PME. A clinical trial lead by the French company MAPREG is currently ongoing aiming to investigate the efficacy of PME in treatment‐resistant depression (NCT03870776). Other companies are also engaging in investigating the potential antidepressant efficacy of PME and its derivatives in MDDs.

## 4 THERAPEUTIC POTENTIAL OF PME IN CDD

CDKL5 deficiency disorder is an X‐linked neurodevelopmental encephalopathy caused by mutations in the *CDKL5* gene characterised by early‐onset, refractory epilepsy, and cognitive and motor developmental delays. As mentioned, CDKL5 is a serine‐threonine kinase that is highly abundant in the brain where it is largely expressed in axonal growth cones, axons, dendrites and spines.^75^, ^76^ The majority of patients are heterozygous females who, as a result of random X‐chromosome inactivation, are chimeras with cells expressing either the mutated or the wild‐type allele.

Various mouse models with inactivation of *Cdkl5* have been generated^77^; *Cdkl5*‐knockout (KO) males and heterozygous (HET) female mice recapitulate most features of the human disorder such as impaired learning and memory, impaired motor control^78^, ^79^, ^80^ and spontaneous seizures.^81^, ^82^

Neuroanatomical analyses performed both in *Cdkl5*‐KO and HET mice have shown a general neurodevelopmental delay characterised by morphological defects such as a reduction in dendritic length and complexity (number of ramifications).^79^, ^80^, ^83^ Improper neuronal architecture was also described *in vitro* in primary cultures of *Cdkl5*‐KO hippocampal neurones, which were found to present an enlarged growth cone area, as well as reduced axon elongation, dendritic length and complexity.^19^ Such defects correlate with the pioneer study of Chen et al,^76^ who showed for the first time that shRNA mediated down‐regulation of *Cdkl5* caused a reduction in axon and dendritic length. Importantly, when overexpressed in cultured neurones, CDKL5 enhanced dendritic elongation in a kinase activity‐dependent manner, suggesting the importance of the catalytic activity for promoting neuronal morphogenesis.^76^

It should be noted that contradictory results have been reported regarding the role of CDKL5 in regulating neuronal morphology. Indeed, a more recent study showed that the absence of CDKL5 impairs dendrite elongation in primary cultures of cortical neurones but not of hippocampal ones,^84^ leaving dendritic complexity unaffected in both types of neurones. This discrepancy with the above reported results may be a result of the different experimental conditions. Although Baltussen et al^84^ used high‐density cultures of green fluoresnce protein‐expressing neurones, low‐density cultures stained with the dendritic marker MAP2 were used in the study of Barbiero et al.^19^

The consequence of pathological mutations in *CDKL5* on dendritic arbourisation was recently examined also in induced pluripotent stem cell‐derived cortical neurones of CDD patients, where an increase in dendritic length and complexity was described.^85^ In line with this result, cortical neurones of post‐mortem tissue from a 5‐year‐old CDD patient displayed an increase in total dendritic length but not dendritic complexity. Interestingly, neurones obtained from a 30‐year‐old patient showed a reduction in both dendritic length and complexity,^85^ possibly indicating that the absence of CDKL5 may lead to a progressive atrophy of the neuronal architecture. This aspect is supported by the study by Tang et al^86^; indeed, surface‐based magnetic resonnace imaging analysis of children with CDD revealed a global volume loss in the cortex and in the subcortical grey matter, which is more pronounced with the progression of the disease. Of relevance, a significant reduction in cortical and hippocampal thickness was described in adult CDD mouse models.^79^

The influence of CDKL5 on neuronal morphology is also reflected in the maturation of dendritic spines. Indeed, several studies have shown that the absence of CDKL5 both *in vitro* and *in vivo* leads to a reduction in the number of mature mushroom‐cup shaped spines.^87^, ^88^, ^89^ In excitatory synapses, loss of CDKL5 leads to a marked depauperation of PSD95 and the surface expression of AMPA receptor subunit GluA2.^85^, ^90^, ^91^, ^92^

The above‐described morphological and molecular defects may be due, at least in part, to the novel and important role of CDKL5 in regulating MT dynamics.^93^ CDKL5 directly phosphorylates different MAPs, among which MAP1S and the +TIP EB2.^84^ Although EB2 is recognised as a *bonafide* substrate of CDKL5, the functional role of this interaction is still unknown.^84^ By contrast, the CDKL5‐mediated phosphorylation of MAP1S on Ser812 was demonstrated to promote MT dynamicity; indeed, CDKL5 expression increased the solubility of MAP1S, thus interfering with its stabilising function of MTs.^84^ Furthermore, as described, CDKL5 associates in a complex with CLIP170 and promotes its MT‐binding conformation.^19^

Interestingly, a phosphoproteomic analysis of CDD patients derived neurones and organoids recently confirmed the involvement of CDKL5 in the regulation of the cytoskeleton and MT network. Indeed, several phosphoproteins, including MAP1A, MAP1B and MAP2, which are associated with MTs, were found to be deregulated.^85^ Although the consequences of the altered phosphorylation in these samples have not yet been investigated, such a finding represents an important starting point for understanding how CDKL5 orchestrates cytoskeleton dynamics and neuronal morphogenesis.

A therapeutic approach aiming at ameliorating the MT‐associated defects has recently been addressed *in vitro*. The treatment with PME, as well as PREG, was found to promote axon length, dendritogensis and the maturation of dendritic spines by increasing the defective expression of PSD95 and GluA2 in *Cdkl5*‐KO neurones.^19^ Although the molecular mechanism of such beneficial effect is still being investigated, it is assumed to depend at least in part on the restoration on CLIP170 functioning. As noted, CLIP170 is less capable of interacting with MTs in CDKL5‐depleted cells.^19^, ^94^ An analogous behaviour was observed in young *Cdkl5*‐KO neurones when axonal outgrowth and elongation occurs. In the growth cone of young *Cdkl5*‐KO neurones, CLIP170 is delocalised from MTs. Such a defect, which implies an improper MT‐binding of CLIP170, appears to be directly associated with the “bundled” spatial conformation of the MT network that was observed in *Cdkl5*‐KO neurones in concomitance with the enlarged area of the axonal growth cone^19^; an overlapping phenotype is caused by the expression of a dominant negative mutant of CLIP170, which interferes with the association of the endogenous protein to MTs.^54^ Importantly, the treatment with PME was found to restore the localisation of CLIP170 with respect to MTs, as well as the morphology of the growth cone.^19^ A similar effect was obtained with PREG, leading to speculate that both molecules promote MT dynamics by inducing the extended conformation of CLIP170, leading to its activation. The same mechanism of action may underlie the effect of PREG and PME on dendritogensis and spine maturation.^19^ The correct homeostasis of MT dynamicity, operated by CLIP170 and other +TIPs, was found to improve dendritic arborisation and the invasion of MTs into dendritic spines. The latter is fundamental to promote spine maturation and synaptic activity.^33^, ^34^, ^55^ The involvement of CLIP170 in CDD‐associated neuronal alterations may offer an explanation for the positive effect of PME and PREG on these defects. However, the recent finding of MAP2 as a CDKL5 target raises the possibility of a parallel action of the two compounds on both of the proteins. Finally, it still remains unknown whether PREG levels are altered in CDD animal models and patients.

### 4.1 Final remarks

Growing evidence suggest a role for endogenous steroids such as PREG with respect to interacting with MT‐associated proteins and in turn modulating MT dynamics. MTs support the development, maintenance and homeostasis of brain functions; insults that hit such a system are implicated in a broad spectrum of neurological pathologies that include psychiatric, neurodegenerative and neurodevelopmental disorders.^22^, ^23^ Albeit, the aetiology of these morbidities is attributable to different factors that span from environment effects to genetic lesions, they are often associated with a dysfunctional MT cytoskeleton. For this reason, the MT system represents a promising target for the development of pharmacological steroid‐derivative treatment. MDDs and CDD, as we have described here, represent two examples of brain pathologies that benefit from the use of the PREG C3 analogue PME. Indeed, by positively affecting MAP functioning and PTMs, PME appears to promote neuronal maturation and connectivity. Although further studies are necessary to clarify the mechanism of action of such compound, in the present review, we have provided evidence indicating that PME is able to promote the association of MAP2 and CLIP170 with the MT lattice, thus ameliorating MT dynamics. Further experiments will certainly be useful to clarify whether PME, similar to PREG, is able to bind both proteins directly. Regarding α‐tubulin PTMs, it is still not clear how treatment with PME prevents their reported alterations in animals models of depression; however, a positive correlation between the MT‐binding of MAP2 and the increase of Acet‐Tub has already been demonstrated.^95^ We can therefore speculate that, by acting on MAP2, PME is able to modify the tubulin code and promote MT dynamics. The role played by α‐tubulin PTMs in CDD is currently under investigation. Additional preclinical and clinical research on the use of PME for the treatment of both MDDs and CDD is ongoing and novel data will be soon disclosed.

## AUTHOR CONTRIBUTIONS

**Isabella Barbiero:** Conceptualisation; Writing – original draft. **Massimiliano Bianchi:** Writing – original draft. **Charlotte Kilstrup‐Nielsen:** Funding acquisition; Writing – review & editing.

### PEER REVIEW

The peer review history for this article is available at https://publons.com/publon/10.1111/jne.13033.
